# Germline Variant Spectrum in Southern Italian High-Risk Hereditary Breast Cancer Patients: Insights from Multi-Gene Panel Testing

**DOI:** 10.3390/cimb46110775

**Published:** 2024-11-15

**Authors:** Valentina Rocca, Elisa Lo Feudo, Francesca Dinatolo, Serena Marianna Lavano, Anna Bilotta, Rosario Amato, Lucia D’Antona, Francesco Trapasso, Francesco Baudi, Emma Colao, Nicola Perrotti, Francesco Paduano, Rodolfo Iuliano

**Affiliations:** 1 Medical Genetics Unit, Renato Dulbecco University Hospital, 88100 Catanzaro, Italy; valentina.rocca@unicz.it (V.R.); elisa.lofeudo@studenti.unicz.it (E.L.F.); francesca.dinatolo@studenti.unicz.it (F.D.); serenamarianna.lavano@studenti.unicz.it (S.M.L.); annabilotta86@gmail.com (A.B.); rosario.amato@unicz.it (R.A.); trapasso@unicz.it (F.T.); baudi@unicz.it (F.B.); colaoemma@unicz.it (E.C.); perrotti@unicz.it (N.P.); 2 Department of Clinical and Experimental Medicine, Campus S. Venuta, University Magna Græcia of Catanzaro, 88100 Catanzaro, Italy; 3 Department of Health Sciences, Campus S. Venuta, University Magna Graecia of Catanzaro, 88100 Catanzaro, Italy; dantona@unicz.it; 4 Stem Cells and Medical Genetics Units, Biomedical Section, Tecnologica Research Institute and Marrelli Health, 88900 Crotone, Italy

**Keywords:** breast cancer (BC), next-generation sequencing (NGS), germline variants, cancer susceptibility genes, founder mutations, genetic heterogeneity

## Abstract

Hereditary breast cancer accounts for 5–10% of all cases, with pathogenic variants in *BRCA1/2* and other susceptibility genes playing a crucial role. This study elucidates the prevalence and spectrum of germline variants in 13 cancer predisposition genes among high—risk hereditary breast cancer patients from Southern Italy. We employed next-generation sequencing (NGS) to analyze 254 individuals selected through genetic counseling. Pathogenic or likely pathogenic variants were identified in 13% (34/254) of patients, with 54% of these variants occurring in non-*BRCA1/2* genes. Notably, we observed a recurrent *BRCA1* c.4964_4982del founder mutation, underscoring the importance of population-specific genetic screening. The spectrum of variants extended beyond *BRCA1/2* to include *PALB2*, *ATM*, *TP53*, *CHEK2*, and *RAD51C*, highlighting the genetic heterogeneity of breast cancer susceptibility. Variants of uncertain significance were detected in 20% of patients, emphasizing the ongoing challenge of variant interpretation in the era of multi-gene panel testing. These findings not only enhance our understanding of the genetic landscape of breast cancer in Southern Italy but also provide a foundation for developing more targeted, population-specific approaches to genetic testing and counseling, ultimately contributing to the advancement of precision medicine in oncology.

## 1. Introduction

Breast cancer remains the most prevalent neoplasia in females worldwide, with approximately 5–10% of cases attributed to hereditary factors. These cases are primarily due to pathogenic variants (PV) in cancer-predisposing genes, most notably the high-penetrance *BRCA1*/*BRCA2* genes, which are inherited in an autosomal-dominant manner [1]. The identification of germline variants in these genes has profound implications for both cancer prevention strategies and targeted therapeutic approaches, making genetic testing an increasingly critical component of breast cancer management.

The landscape of genetic testing for breast cancer susceptibility has evolved significantly over the past two decades. Testing was initially limited to BRCA*1*/*2* genes only, but our understanding of breast cancer genetics has expanded dramatically.

Current evidence suggests that approximately 50% of the pathogenic variants identified are associated with additional predisposition genes linked to varying levels of breast cancer risk [2,3]. While *BRCA1* and *BRCA2* remain the most well-known high-penetrance genes, others, such as *ATM*, *BARD1*, *CHEK2*, *PALB2*, *RAD51C*, and *RAD51D*, are now recognized as conferring moderate to high risk for breast cancer [3]. In a previously published study, we observed that among the 13 breast cancer individuals with positive germline pathogenic variant findings, the majority had variants in the *BRCA1*/*2* genes, while the remaining individuals had variants in other high- or moderate-risk genes, such as *PALB2*, *TP53*, *ATM*, and *CHEK2* [2].

Recent landmark studies have significantly advanced our understanding of breast cancer susceptibility genes. Two comprehensive case-control analyses by Dorling et al. and Hu et al. evaluated the associations between variants in 34 and 28 putative cancer susceptibility genes, respectively, and breast cancer risk [4,5]. These studies definitively established that pathogenic variants in eight canonical genes, including *BRCA1*, *BRCA2*, *PALB2*, *BARD1*, *RAD51C*, *RAD51D*, *ATM*, and *CHEK2*, demonstrate significant association with increased breast cancer susceptibility. The international study by Dorling et al. additionally identified an association with variants in *MSH6* [4], while the US-based study by Hu et al. identified an association with variants in *CDH1* [5]. Importantly, these studies revealed distinct patterns in the distribution of mutations between breast cancer cases and controls. Mutations in the high-risk genes *BRCA1*, *BRCA2*, and *PALB2* were more prevalent in cases, while mutations in the moderate-risk genes *CHEK2* and *ATM* showed higher prevalence in controls [6].

This differential distribution has important clinical implications, particularly as the increasing detection of moderate-risk gene variants through panel testing necessitates careful counseling of unaffected women regarding cancer risks and management options. Together, these complementary studies have significantly clarified the genetic landscape of hereditary breast cancer predisposition, providing valuable insights that inform the development of more comprehensive and personalized risk assessment and prevention strategies [6].

The advent of next-generation sequencing (NGS) technologies has revolutionized the field of genetic testing, enabling the simultaneous analysis of multiple genes through multi-gene panels. This technological leap has facilitated a more comprehensive assessment of cancer predisposition genes, including those conferring high-, moderate-, and low-penetrance risks. The shift from single-gene testing to multi-gene panels has significantly enhanced our ability to identify individuals at increased risk and tailor prevention and treatment strategies accordingly [7,8,9,10,11].

The identification of pathogenic (VP) or likely pathogenic (LP) variants in these susceptibility genes has far-reaching implications for patient care. It enables improved follow-up of carrier patients by adopting two primary strategies: early detection using enhanced imaging protocols and targeted therapeutic approaches [12,13,14]. This is particularly evident in the development and implementation of poly (ADP-ribose) polymerase inhibitors that have led to improvements in breast and ovarian cancer treatments. Moreover, germline testing results now serve as predictive biomarkers, guiding treatment decisions and potentially improving outcomes for patients with specific genetic profiles [13,14,15].

Central to the effective implementation of these advances is genetic counseling, which has emerged as a critical component in the management of hereditary breast cancer risk. This essential clinical activity involves collecting detailed personal and family health histories to identify individuals who may benefit from genetic testing [15,16,17].

The process of genetic counseling has become increasingly sophisticated, incorporating advanced risk assessment tools. During these sessions, the likelihood of carrying pathogenic variants in moderate-to-high-risk genes can be calculated using complex cancer risk models, such as the Breast and Ovarian Analysis of Disease Incidence and Carrier Estimation Algorithm (BOADICEA) [18,19]. These models integrate multiple factors, including family history, tumor pathology, and demographic variables, to provide a more accurate assessment of an individual’s genetic risk.

The rapid integration of multi-gene panel testing into clinical practice has also brought new challenges. While these panels offer a more comprehensive genetic analysis, they often generate results of uncertain significance, particularly in genes whose clinical involvement in breast cancer is less established [9,10]. This underscores the need for ongoing research to clarify the clinical implications of variants in lesser-known cancer susceptibility genes and to provide evidence-based management recommendations.

In this context, our study aims to elucidate the prevalence and spectrum of germline variants in 13 cancer susceptibility genes among a cohort of high-risk hereditary breast cancer patients from Southern Italy. The Italian population exhibits significant genetic heterogeneity, with Southern Italy, particularly the Calabria region, representing a distinctive genetic isolate characterized by historical isolation and documented founder effects. Previous studies in this population have identified significant founder mutations, including the *BRCA1* c.4964_4982del variant, highlighting the importance of population-specific genetic analyses in hereditary breast cancer risk assessment [20,21].

By employing a comprehensive NGS panel, we seek to characterize the genetic landscape of hereditary breast cancer in this population. This approach allows us to move beyond the traditional focus on *BRCA1/2* genes and explore the contribution of other high- and moderate-risk genes to breast cancer susceptibility in this specific population [11].

Additionally, we aim to evaluate the efficacy of the BOADICEA model in identifying patients suitable for genetic testing. This assessment is crucial for refining and validating risk prediction tools, which play a vital role in the appropriate selection of candidates for genetic testing and subsequent clinical management.

Our findings contribute to the growing body of knowledge on the genetic basis of breast cancer susceptibility and have significant implications for refining genetic testing strategies in diverse populations [7,8,9,10,11,22]. By providing insights into the genetic profile of breast cancer patients in Southern Italy, this study intends to promote the development of more targeted and population-specific approaches to genetic testing and counseling; it underscores the importance of considering ethnic and geographic variations in genetic risk profiles, which are essential for the effective implementation of precision medicine approaches in oncology.

## 2. Materials and Methods

### 2.1. Patients Selection

A total of 254 patients were selected after genetic counseling between September 2019 and October 2023 for the NGS genetic test according to the family and personal criteria established by the Associazione Italiana di oncologia Medica (AIOM, 2023) [23].

Patient recruitment was conducted at the Medical Genetics Unit of Renato Dulbecco University Hospital in Catanzaro (Italy), a specialized facility for genetic testing and counseling. Patients were referred through a regional clinical network including the Dulbecco Breast Cancer Unit, Regional Oncology Units, and primary care physicians. While primarily serving the province of Catanzaro, the center also received referrals from the neighboring provinces of Crotone, Cosenza, Vibo Valentia, and Reggio Calabria, representing a comprehensive sampling of the Calabrian population.

The patient’s cancer history (clinical diagnosis, age of first cancer, histological stage) and the number of affected relatives were evaluated through genetic counseling.

The inclusion criteria for the NGS genetic test were:

(1) Individuals with both breast and ovarian cancers;
(2) Individuals with breast cancer ≤40 years;
(3) Individuals with triple-negative breast cancer (any age);
(4) Individuals with bilateral breast cancer ≤50 years;
(5) Individuals with male breast cancer.

This study was approved by “Comitato Etico Territoriale Regione Calabria” (Protocol n. 128 of 23 April 2024) and conducted according to the principles of the Declaration of Helsinki. Before NGS panel testing, a consent form for the donation of human materials was completed by the patients.

### 2.2. DNA Extraction and NGS

Genomic DNAs from patients were extracted from blood samples using the NLM DNA extraction kit (Nuclear Laser Medicine) following the manufacturer’s instructions. The eluted DNA concentrations were determined using a Qubit 3.0 fluorometer (Thermo Fisher Scientific). We designed Ion Ampliseq On-Demand panels to explore using Ion Torrent, the mutational status of the most frequently altered genes in breast cancer patients.

This panel contained 409 primer pairs in two pools, allowing for a comprehensive sequencing of 13 genes: *BRCA1*, *BRCA2*, *ATM*, *PALB2*, *TP53*, *CHEK2*, *MLH1*, *MSH2*, *MSH6*, *PMS2*, *BRIP1*, *RAD51C*, and *RAD51D.* Briefly, library preparation was performed automatically on the Ion Chef platform using the Ion AmpliSeq Chef Solutions DL8 Kit (Thermo Fisher Scientific, Waltham, MA, USA). This was followed by clonal amplification, which was also performed automatically on the Ion Chef Platform using the Ion 510 & Ion 520 & Ion 530 Kit-Chef (Thermo Fisher Scientific). Finally, the prepared sequencing templates were sequenced using Ion 510™ Chips and the Ion S5 System’s Kit (Thermo Fisher Scientific, Waltham, MA, USA), according to the manufacturer’s guidelines.

### 2.3. Sanger Sequencing

Genomic DNAs were amplified by PCR using the forward and reverse primers binding to the selected exons of *BRCA1*, *BRCA2*, *ATM*, *PALB2*, *TP53*, *CHEK2*, *MLH1*, *MSH2*, *MSH6*, *PMS2*, *BRIP1*, *RAD51C*, and *RAD51D* genes. Amplicons were bidirectionally sequenced using Big Dye Terminator 1.1 on a SeqStudio Genetic Analyzer (Thermo Fisher Scientific, Waltham, MA, USA).

### 2.4. Bioinformatics Analysis

After sequencing, the generated data were initially processed on the Ion Torrent Suite software version 5.12 (Ion Torrent; Thermo Fisher Scientific, Inc.) to generate filtered sequence reads and remove poor signal-profile reads. The resulting reads were then aligned to the hg19 (GRCh37) genome using the reference genome sequence that targets the 13 genes. BAM and VCF data were also analyzed by the Integrative Genomic Viewer tool (IGV). Germinal variants that had an allele frequency below 0.01 based on allele frequencies found in GnomAD were the ones retained for further investigation. Variants were classified using sequence variation databases like ClinVar and LOVD. The unknown detected variants in our study were classified following the American College of Medical Genetics and Genomics (ACMG) guidelines [24]. We used this data for descriptive statistics. The bioinformatic analysis pipeline used in this study did not include CNV detection algorithms.

## 3. Results

**Figure 1:**
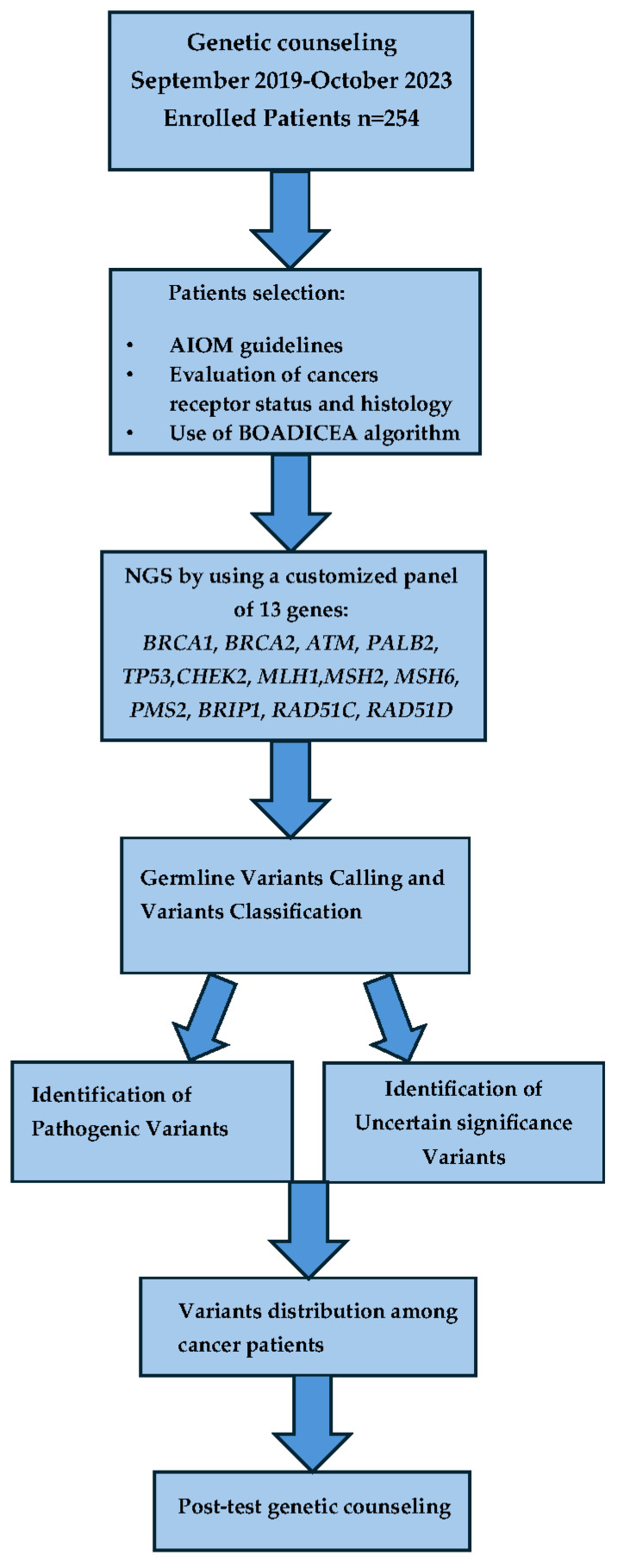
Study flow chart.

The study flow chart is reported in Figure 1. Between September 2019 and October 2023, a total of 254 breast cancer patients received genetic counseling that was performed to evaluate the patient’s cancer history, including clinical diagnosis, age of cancer diagnosis, histological stage, molecular subtype, and family history of cancer according to the family and personal criteria established by the AIOM criteria, based on the recommendations of the National Comprehensive Cancer Network.

The clinical characteristics of the patients are summarized in Table 1.

The breast cancer patient cohort included 242 women and 12 men; the mean age of diagnosis was 51 years (range, 25–91). Approximately 67% of the patients (*n* = 171) had invasive ductal carcinoma, 8% had invasive lobular carcinoma, 3% had in situ ductal carcinoma, and 4% had other cancer types.

**Figure 2:**
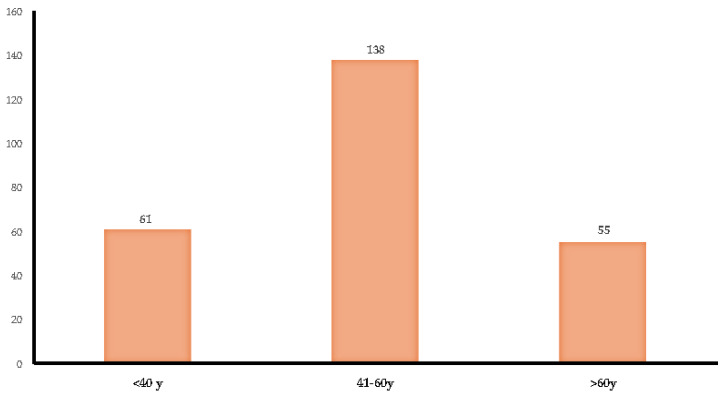
Age of diagnosis of breast cancer among patients.

A total of 61/254 (24%) patients received a breast cancer diagnosis before the age of 40 years, 138/254 (54%) received a diagnosis between 41 and 60 years, and 55/254 (22%) received a breast cancer diagnosis after the age of 60 years (Figure 2).

The Breast and Ovarian Analysis of Disease Incidence and Carrier Estimation Algorithm “BOADICEA” is a risk prediction model that calculates the probability of carrying rare PVs in moderate-to-high-risk genes in breast cancer patients and estimates the future risks of developing breast or ovarian cancer. BOADICEA uses data on family cancer history, screening for variants in high-risk genes, tumor pathology, and basic demographic variables like year of birth and country. BOADICEA V5’s newest version includes the effects of pathogenic variants (PVs), not restricted to *BRCA1* and *BRCA2* genes but also in *PALB2*, *CHEK2*, *ATM*, and *BARD1* for the breast cancer model and *RAD51D*, *RAD51C*, and *BRIP1* for the ovarian cancer model [23,25].

The likelihood of carrying PVs for 222 breast cancer patients was calculated by the BOADICEA model based on personal and family cancer history, histology, mammographic density, molecular subtype, hormonal risk factors, and lifestyle using a 10% pretest probability threshold. Patients were stratified into two groups: those with BOADICEA scores > 10% (high risk, *n* = 122) and those with scores ≤ 10% (low risk, *n* = 100). The high-risk group represented individuals with an elevated probability of carrying pathogenic variants in breast cancer susceptibility genes, while the low-risk group included those with a lower likelihood. This stratification was based on the established 10% threshold for genetic testing recommendations (Figures S1 and S2).

### 3.1. Variants Distribution

**Figure 3:**
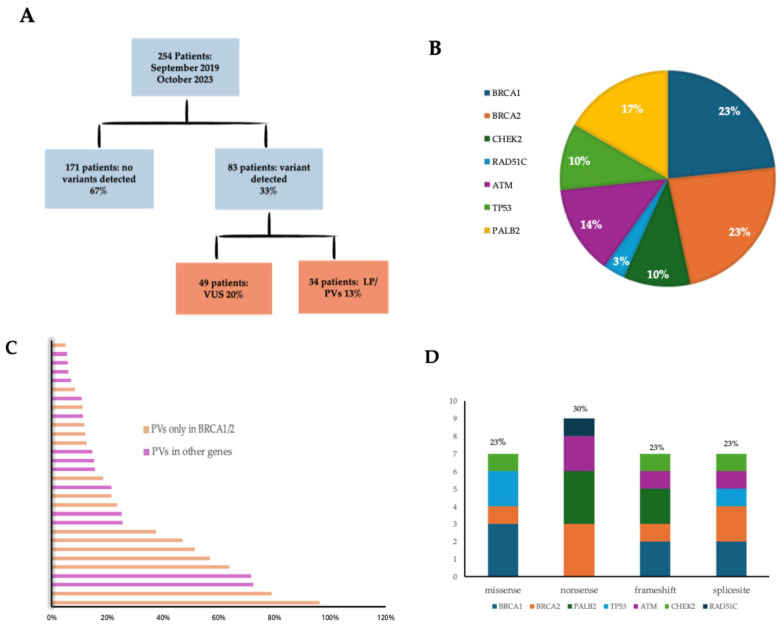
(**A**) Results of NGS panel testing. (**B**) Numbers of deleterious variants by gene. (**C**) Patients’ BOADICEA scores that have deleterious variants. (**D**) Distribution of pathogenic variants by effects on genes.

NGS germline testing revealed uncommon variants in 83 of 254 patients (33%). No variants were detected in 171 patients (67%), while 49 patients had VUSs (20%), and 34 (13%) had LP/P variants (Figure 3A).

A total of 88 variants were detected, and 30 of 88 (34%) were LP/PV variants, and 14/30 (47%) were in the *BRCA1/2* genes, whereas 16/30 (53%) were in other high- or moderate-risk genes, including *TP53*, *PALB2*, *ATM*, *CHEK2*, and *RAD51C.* The pathogenic variants were 25/30 (83%), and the likely pathogenic variants were 5/30 (17%).

Concerning the germline LP/PV identified among genes, seven of thirty (23%) were in *BRCA1*, seven of thirty (23%) were in *BRCA2*, five of thirty (17%) were in *PALB2*, four of thirty (14%) were in *ATM*, three of thirty (10%) were in *CHEK2*, three of thirty (10%) were in *TP53*, and one of thirty (3%) was in *RAD51C* (Figure 3B), and the BOADICEA scores of the patients who tested positive in those were calculated, as shown in Figure 3C.

The complete list of LP/P variants is listed in Table 2. Seven of thirty (23%) LP/PVs were missense, nine of thirty were non-sense variants (30%), seven of thirty were frameshift variants (23%), and seven of thirty were splicing variants (23%) (Figure 3D).

### 3.2. Variants of Uncertain Significance

We detected a total of 58/88 (66%) variants of uncertain significance (VUSs). One VUS was identified in 49 patients (20%). In three patients, we detected two VUSs, and eight patients with a VUS also had a deleterious mutation. Most of the VUSs were missense variants (*n* = 54), one was an intronic variant (*MSH6*: c.4001+42_4001+45dup), one was an in-frame indel (*BRCA2*: c.1216_1219delinsACCG), one was a synonymous variant (*BRCA1*: c.1881C>G), and one was 5′-UTR (*CHEK2*: c.-4C>T).

Among the 49 patients that did not have other LP/PVs, five carried VUSs in the *BRCA1* gene, four in *BRCA2*, four in *BRIP1*, nine in *ATM*, six in *CHEK2* (four patients had the same two variants), five in *PMS2*, five in *PALB2*, six in *MSH6*, one in *TP53*, and one in *RAD51D*. Three patients were carriers of two VUSs, i.e., patient 979/21 had VUSs in the *CHEK2* and *MLH1* genes, patient 267/22 had both VUSs in the *ATM* gene, and 309/23 had VUSs in the *MSH6* and *PMS2* genes). All VUSs identified are listed in Table S1.

## 4. Discussion

This study, using next-generation sequencing (NGS) with a multi-gene panel assay in 254 high-risk hereditary breast cancer patients from Southern Italy, provided valuable insights into the genetic landscape of breast cancer susceptibility, revealing implications for genetic testing strategies and clinical management.

Our analysis revealed that 13% of patients carried germline pathogenic variants in 7 of the 13 predisposition genes tested. Notably, while *BRCA1/2* germline variants accounted for 46% (23% in *BRCA1* and 23% in *BRCA2*) of these pathogenic changes, a substantial 54% were found in other breast cancer predisposition genes [2].

This distribution underscores the importance of comprehensive multi-gene panel testing beyond *BRCA1/2*, aligning with recent trends in genetic screening for hereditary breast cancer [7,8,9,10,11].

Our results demonstrate that the utilization of technologies enabling the rapid generation of increasing volumes of data (big data) presents a significant advantage in both research and diagnostics, allowing for an increased number of diagnoses and identification of at-risk individuals.

The spectrum of pathogenic variants identified in our cohort reflects the genetic heterogeneity of breast cancer susceptibility. We observed mutations across several high- and moderate-penetrance genes, including *PALB2*, *ATM*, *TP53*, *CHEK2*, and *RAD51C*.

Our analysis of moderate-penetrance genes revealed the presence of pathogenic variants, particularly in *CHEK2* and *ATM*. We identified three *CHEK2* variants, including the pathogenic c.1100del variant, which is known to confer a greater than twofold increased breast cancer risk [63]. These findings align with previous studies suggesting that *CHEK2* and *ATM* mutations increase breast cancer risk [72,73], underscoring their clinical relevance in breast cancer susceptibility.

The expansion of analyzed genes and the identification of variants in genes with a varying penetrance introduce emerging challenges in patient management. The difficulty in formulating specific guidelines results in potentially divergent interpretations and recommendations for patients and their families. In our cohort, we identified cases where multiple variants were associated in the same individual (for example, between *ATM* and *CHEK2*), suggesting a possible cumulative effect with altered risk in carriers. Similarly, these genetic conditions could underlie a pleiotropic effect, explaining the presence of atypical family histories that deviate from BOADICEA predictions.

Concurrently, the identification of variants in lower-penetrance genes has implications for surveillance, sometimes necessitating the exclusion of their presence as incidental findings. Our results confirm the need to define specific criteria for interpreting the clinical significance of constitutional genetic variants, contributing to their collection in joint databases [74].

The presence of mutations in genes other than *BRCA1/2* impacts prognosis and the therapeutic approach. For instance, the field of targeted therapy, which originated with the use of PARP inhibitors in tumors with *BRCA* gene mutations, is expanding to new genes, offering the possibility of selective therapies for carriers of mutations in other genes, such as *PALB2* and others present in our cohort.

Interestingly, we identified pathogenic variants in *TP53* (c.376-1G>A and c.451C>G) in two patients with triple-negative breast cancer (TNBC). This finding is consistent with previous reports indicating that *TP53* mutations are present in up to 80% of TNBCs [72], highlighting the potential role of *TP53* testing in this breast cancer subtype.

The detection of mutations in this gene, traditionally associated with the rare Li–Fraumeni Syndrome, is increasing. We are also observing an increase in the occurrence of different tumors associated with breast cancer (frequently affecting the colon and stomach). The use of multi-gene panels, applied to a broader spectrum of tumors, is leading to a higher detection of genetic mutations, suggesting the presence of atypical associations of a wider and more heterogeneous spectrum of tumors. As accredited guidelines do not account for the heterogeneity of familial cases, it is necessary to improve the definition of the phenotype associated with mutations in these genes. The surveillance protocol in such cases must adapt to the clinical characteristics of the patient and the family, impacting the complexity of the patient’s clinical management.

Our study also provided insights into male breast cancer genetics, an often-understudied area. Among the 12 male breast cancer patients in our cohort, we identified pathogenic variants in *CHEK2* (c.846+1G>C) and *PALB2* (c.758dup) [75]. These findings emphasize the importance of genetic testing in male breast cancer patients and suggest that genes beyond *BRCA2* can play a role in male breast cancer susceptibility. Notably, current NCCN guidelines have expanded to address these clinical implications for gender-diverse populations.

The mutational spectrum of hereditary breast cancer exhibits substantial geographic heterogeneity across European populations [76]. Analysis of regional variation in Southern Italy revealed distinctive patterns of founder mutations, particularly in *BRCA1/2* genes. Large-scale studies from Apulia demonstrated that the *BRCA1* c.5266dupC variant represents 54.9% of *BRCA1* carriers among 2.026 hereditary breast and ovarian cancer patients [77]. In Sicily, an investigation of 1.967 subjects identified the *BRCA1* c.4964_4982del19 variant as a predominant mutation, occurring in 18 families and accounting for 13% of *BRCA*-positive carriers [78]. Our Calabrian cohort corroborated these regional patterns, with the same c.4964_4982del19 variant representing 16.6% of *BRCA1/2* mutations and 30% of *BRCA1* carriers.

The Calabrian founder mutation is distinguished by a phenotype in which the appearance of breast and ovarian cancer (BOC) prevails, and its identification is particularly important for surveillance and the choice of risk-reduction strategies in carrier family members [20,79].

The use of the BOADICEA model in our study provided additional insights into risk prediction. We found that 55% of patients had a BOADICEA score > 10%, classified as high risk for carrying pathogenic variants in breast cancer susceptibility genes. This highlights the utility of risk prediction models in identifying individuals who may benefit most from genetic testing [23,25].

Our study also revealed challenges associated with variant interpretation. We identified variants of uncertain significance (VUSs) in 20% of patients, a finding consistent with the challenges faced in the era of multi-gene panel testing [9,10]. This underscores the need for ongoing research to clarify the clinical implications of these variants and improve variant classification.

The majority of VUSs identified in our cohort were missense variants (54/58, 93.1%), with most *BRCA1/2* variants located in coldspot regions, genomic areas showing a lower frequency of pathogenic variants and greater tolerance to amino acid substitutions [80]. However, three variants identified in our study deserve particular attention for their potential pathogenicity. Two *CHEK2* variants (c.911T>C and c.1160C>T) were recently characterized as functionally impaired in a comprehensive analysis of *CHEK2* missense variants [81]. Additionally, the *ATM* variant c.8560C>T (p.Arg2854Cys), located in the kinase domain, showed deleterious in silico predictions and increased frequency in cancer cohorts, compared to controls [82]. These findings highlight the importance of ongoing variant classification efforts and functional studies in determining the clinical significance of VUSs.

The application of multi-gene panels accentuates ethical and communication issues in counseling settings, impacting the psychological evaluation of receiving a positive test for a lower-penetrance gene or the presence of a VUS. It is crucial to inform patients during pre-test counseling about the possibility of identifying incidental variants or those without known significance. Similarly, in post-test counseling, when discussing recommendations for screening or prophylactic measures, it is important to decide whether and how to communicate the presence of variants that do not have a certain impact on tumor risk.

In conclusion, our study provides a comprehensive evaluation of the genetic basis of hereditary breast cancer in a Southern Italian population. The significant proportion of pathogenic variants identified in non-*BRCA1*/*2* genes (54%) supports the clinical utility of multi-gene panel testing in this population.

These results have important implications for genetic counseling, testing strategies, and clinical management of high-risk individuals in this population. They support a more comprehensive approach to genetic testing that goes beyond *BRCA1/2* and takes into account the contributions of moderate-penetrance genes and population-specific variants.

Our study also contributes to the systematic and centralized collection of *BRCA* variants, aiming for better classification and strengthening genotype–phenotype associations. The need to overcome associated clinical challenges also emerges. In particular, it is increasingly important to adopt national reference diagnostic and treatment protocols (PDTA) and to have the opportunity to manage patients with tumor susceptibility in a multidisciplinary manner.

Future research should focus on further characterizing the clinical implications of variants in less-well-studied genes and clarifying the significance of VUSs. Additionally, prospective studies with larger, diverse cohorts could help validate these findings and potentially uncover additional population-specific genetic factors.

Advances in breast cancer genomics are rapidly reshaping clinical paradigms. Our findings underscore the urgent need to refine genetic testing strategies, recalibrate risk models, and tailor preventive interventions. This adaptive approach is crucial for realizing the promise of precision oncology in breast cancer management.

## Figures and Tables

**Table 1:** Characteristics of the BC cohort. TNBC, triple-negative breast cancer.

Characteristics	No. (%)
Sex assigned at birth Female Male	242 (95%) 12 (5%)
Age of Diagnosis, Media (Range)	51 (25–91)
Histologic diagnosis Invasive ductal carcinoma Invasive lobular carcinoma In situ ductal carcinoma Others Unknown	171 (67%) 20 (8%) 6 (4%) 11 (4%) 46 (18%)
Subtype Triple Negative (TNBC) Others Unknown	45 (18%) 151 (60%) 58 (22%)

**Table 2:** Germline likely pathogenic (LP) and pathogenic variants (PV) identified in our study; NR, not reported.

N.	Gene	Variant (HGVS) GRCh37/hg19	dbSNP	Type of Variant	ClinVar/ACMG Classification	Ref
1	*ATM*	Chr11:g.108186742C>T c.6100C>T (p.Arg2034Ter)	rs532480170	non-sense	Pathogenic	[26,27]
2	*ATM*	Chr11:g.108190781-108190782dup c.6450dup (p.Arg2151GlnfsTer10)	rs2136222670	frame-shift	Pathogenic	[28]
3	*ATM*	Chr11:g.108205837G>T c.8151+1G>T	NR	splice-site	Likely Pathogenic	NR
4	*ATM*	Chr11:g.108235935C>T c.8977C>T (p.Arg2993Ter)	rs770641163	non-sense	Pathogenic	[29]
5	*BRCA1*	Chr17:g.41267741A>G c.134+2T>C	rs80358131	splice-site	Pathogenic	[30]
6	*BRCA1*	Chr17:g.41267741A>G c.134+2T>G	rs80358131	splice-site	Pathogenic	NR
7	*BRCA1*	Chr17:g.41258504T>G c.181T>G (p.Cys61Gly)	rs28897672	missense	Pathogenic	[31,32]
8	*BRCA1*	Chr17:g.41258473G>C c.212G>C (p.Arg71Thr)	rs80356913	missense	Pathogenic	[33,34]
9	*BRCA1*	Chr17:g.41246187- 41246188del c.1360_1361del (p.Glu453_Ser454insTer)	rs80357969	frame-shift	Pathogenic	[21,35]
10	*BRCA1*	Chr17:g.41222949-41222967del c.4964_4982del (p.Ser1655TyrFsTer16)	rs80359876	frame-shift	Pathogenic	[36,37]
11	*BRCA1*	Chr17:g.41215920C>A c.5123C>A (p.Ala1708Glu)	rs28897696	missense	Pathogenic	[38,39]
12	*BRCA2*	Chr13:g.32907285T>G c.1670T>G (p.Leu557Ter)	rs80358452	non-sense	Pathogenic	[40,41]
13	*BRCA2*	Chr13:g.32907526T>A c.1909+2T>A	rs876658577	splice-site	Likely Pathogenic	[2]
14	*BRCA2*	Chr13:g.32912623- 32912624insTGAGGA c.4131_4132insTGAGGA (p.Thr1378Ter)	rs80359429	Non-sense	Pathogenic	[42,43]
15	*BRCA2*	Chr13:g.32914401C>A c.5909C>A (p.Ser1970Ter)	rs80358824	non-sense	Pathogenic	[44,45]
16	*BRCA2*	Chr13:g.32914894-32914898del c.6405_6409del (p.Asn2135LysFsTer3)	rs80359584	frame-shift	Pathogenic	[46]
17	*BRCA2*	Chr13:g.32921033G>A c.7007G>A (p.Arg2336His)	rs28897743	missense	Pathogenic	[47,48]
18	*BRCA2*	Chr13:g.32944695G>A c.8487+1G>A	rs81002798	splice-site	Pathogenic	[49,50]
19	*CHEK2*	Chr22:g.29121087T>C c.470T>C (p.Ile157Thr)	rs17879961	missense	Likely Pathogenic	[51,52]
20	*CHEK2*	Chr22:g.29105993C>A c.846+1G>C	rs864622149	splice-site	Likely Pathogenic	[53,54]
21	*CHEK2*	Chr22:g.29091857del c.1100del (p.Thr367MetfsTer15)	rs555607708	frame-shift	Pathogenic	[55,56]
22	*PALB2*	Chr16:g.23649207-23649210del c.172_175del (p.Gln60ArgFsTer7)	rs180177143	frame-shift	Pathogenic	[57,58,59]
23	*PALB2*	Chr16:g.23647108-23647109dup c.758dup (p.Ser254IlefsTer3)	rs515726126	frame-shift	Pathogenic	[60,61]
24	*PALB2*	Chr6:g.23646419C>G c.1448C>G (p.Ser483Ter)	rs1057520736	non-sense	Pathogenic	NR
25	*PALB2*	Chr16:g.23646416A>T c.1451T>A (p.Leu484Ter)	rs786203714	non-sense	Pathogenic	[62,63]
26	*PALB2*	Chr16:g.23641107C>T c.2368C>T (p.Gln790Ter)	rs886039480	non-sense	Pathogenic	[64,65]
27	*RAD51C*	Chr17:g.56780562C>T c.577C>T (p.Arg193Ter)	rs200293302	non-sense	Pathogenic	[66,67]
28	*TP53*	Chr17:g.7578555C>T c.376-1G>A	rs868137297	splice-site	Pathogenic	[68]
29	*TP53*	Chr17:g.7578479G>C c.451C>G (p.Pro151Ala)	rs28934874	missense	Pathogenic	[69,70,71]
30	*TP53*	Chr17:g.7578467T>C c.463A>C (p.Thr155Pro)	rs772683278	missense	Likely Pathogenic NR	

## Data Availability

The original contributions presented in the study are included in the article/Supplementary Material, further inquiries can be directed to the corresponding authors.

## Supplementary Materials

The following supporting information can be downloaded at: https://www.mdpi.com/article/10.3390/cimb46110775/s1, Figure S1: Cancer risk score > 10%; Figure S2: Cancer risk score < 10%; Table S1: List of VUS identified in this study; References [2,58,59,63,67,75,80,83,84,85,86,87,88,89,90,91,92,93,94,95,96,97,98,99,100,101,102,103,104,105,106,107,108,109,110,111,112,113,114,115] are cited in the supplementary materials.
