# Supplementary material for: Germline Variant Spectrum in Southern Italian High-Risk Hereditary Breast Cancer Patients: Insights from Multi-Gene Panel Testing
Source: Curr Issues Mol Biol. 2024 Nov 15;46(11):13003–20. doi: 10.3390/cimb46110775 (PMC11592649; doi:10.3390/cimb46110775)
Supplement: Supplementary file 1 [file cimb-46-00775-s001.zip › cimb-3298387-supplementary.pdf]

Supplementary Figure S1

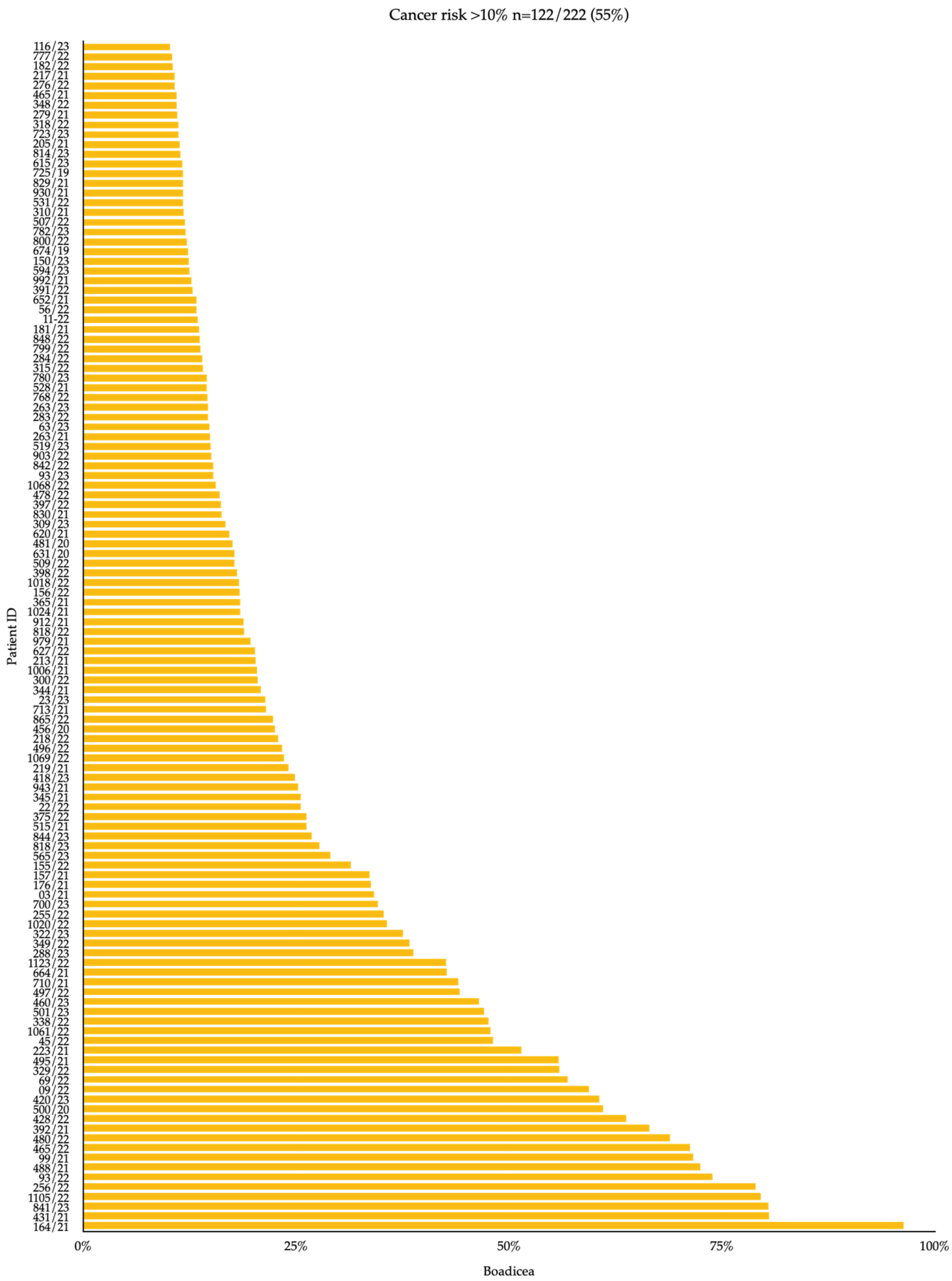

Supplementary Figure S2

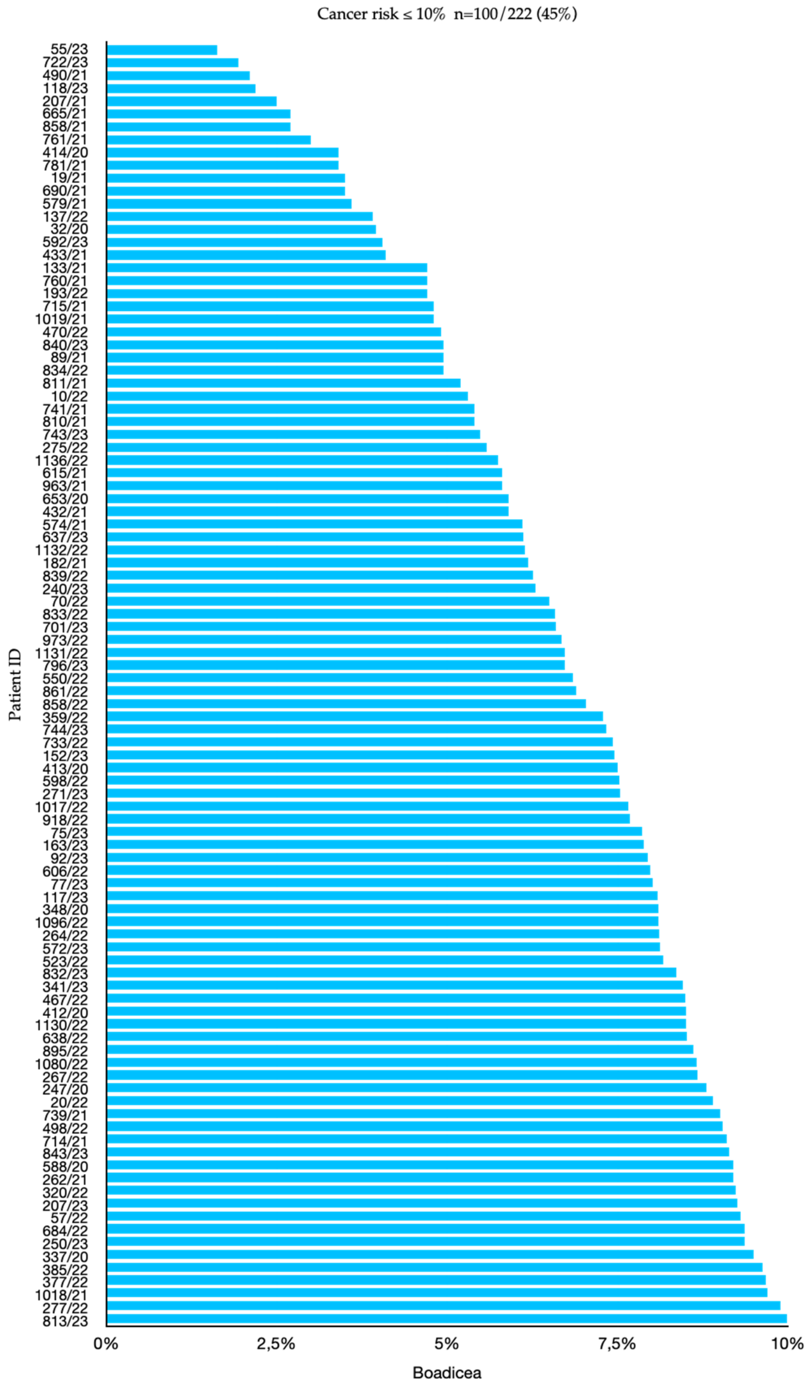

Supplementary Table S1: List of VUS identified in this study.

	Gene	Variant (HGVS) GRCh37/hg19	dbSNP	Type of Variant	ClinVar/ACMG classification	Ref
1	***ATM***	Chr11:g.108099919A>G c.200A>G (p.Tyr67Cys)	rs754033733	missense	VUS	NR
2	***ATM***	Chr11:g.108117767A>G c.978A>C (p.Ile326Met)	rs1555068424	missense	VUS	[1]
3	***ATM***	Chr11:g.108121708G>T c.1516G>T (p.Gly506Cys)	rs587779816	missense	VUS	[2]
4	***ATM***	Chr11:g.108122571G>C c.1615G>C (p.Val539Leu)	NR	missense	VUS	NR
5	***ATM***	Chr11:g.108122604A>G c.1648A>G (p.Ile550Val)	rs202144949	missense	VUS	[3]
6	***ATM***	Chr11:g.108142010A>G c.2954A>G (p.Asp985Gly)	rs864622159	missense	VUS	NR
7	***ATM***	Chr11:g.108143261A>G c.3080A>G (p.His1027Arg)	rs786204217	missense	VUS	[4]
8	***ATM***	Chr11:g.108150289C>T c.3356C>T (p.Ala1119Val)	rs778882461	missense	VUS	NR
9	***ATM***	Chr11:g.108160398C>T c.4306C>T (p.His1436Tyr)	rs544891616	missense	VUS	[4]
10	***ATM***	Chr11:g.108180900A>G c.5776A>G (p.Thr1926Ala)	rs781448339	missense	VUS	NR
11	***ATM***	Chr11:g.108186568T>C c.6025T>C (p.Tyr2009His)	rs199586999	missense	VUS	NR
12	***ATM***	Chr11:g.108200949T>C c.7316T>C (p.Val2439Ala)	rs776266049	missense	VUS	[5,6]
13	***ATM***	Chr1:g.1108201108T>G c.7475T>G (p.Leu2492Arg)	rs56399857	missense	VUS	[7]
14	***ATM***	Chr11:g.108216611C>T c.8560C>T (p.Arg2854Cys)	rs201958469	missense	VUS	[8,9]
15	***BRCA1***	Chr17:g.41246298T>C c.1250A>G (p.Asn417Ser)	rs80357113	missense	VUS	[10]
16	***BRCA1***	Chr17:g.41246204C>G c.1344C>G (p.His448Gln)	rs1395644015	missense	VUS	[11]

17	***BRCA1***	Chr17:g.41245667G>C c.1881C>G (p.Val627Val)	rs80356838	synonymous	VUS	NR
18	***BRCA1***	Chr17:g.41243928T>A c.3620A>T (p.Lys1207Met)	rs1555587402	missense	VUS	NR
19	***BRCA1***	Chr17:g.41203100G>T c.5312C>A (p.Pro177His)	rs80357025	missense	VUS	NR
20	***BRCA2***	Chr13:g.32905126C>G c.752C>G (p.Thr251Arg)	rs587781513	missense	VUS	[12]
21	***BRCA2***	Chr13:g.32906831-32906834delinsACCG c.1216_1219delinsACCG (p.Ala406_Gln407delinsThrGl)	rs886037742	Indel in frame	VUS	NR
22	***BRCA2***	Chr13:g.32912201G>C c.3709G>C (p.Ala1237Pro)	rs398122770	missense	VUS	NR
23	***BRCA2***	Chr13:g.32913659A>G c.5167A>G (pThr1723Ala)	rs80358742	missense	VUS	[13]
24	***BRIP1***	Chr17:g.59926582A>C c.415T>G (p.Ser139Ala)	rs202072866	missense	VUS	[14]
25	***BRIP1***	Chr17:g.59924539C>T c.550G>A (p.Asp184Asn)	rs201047375	missense	VUS	[15,16]
26	***BRIP1***	Chr17:g.59871062C>T c.1369G>A (p.Val457Ile)	rs748221377	missense	VUS	NR
27	***BRIP1***	Chr17:g.59763205T>C c.2897A>G (p.Lys966Arg)	NR	missense	VUS	NR
28	***CHEK2***	Chr22: g.29130713G>A c.-4C>T	rs374938148	5' UTR variant	VUS	[17]
29	***CHEK2***	Chr22:g.29130651T>C c.59A>G (p.Gln20Arg)	rs753257724	missense	VUS	[18]
30	***CHEK2***	Chr22:g.22:29107902C>G c.787G>C (p.Glu263Gln)	rs587780188	missense	VUS	[19,20]
31	***CHEK2***	Chr22:g.29099495T>G c.906A>C (p.Glu302Asp)	rs587780190	missense	VUS	[16]
32	***CHEK2***	Chr22:g.29095923A>G c.911T>C (p.Met304Thr)	rs587782033	missense	VUS	[21,22]
33	***CHEK2***	Chr22:g.29091797G>A c.1160C>T (p.Thr387Ile)	rs587780168	missense	VUS	[23,24]
34	***CHEK2***	Chr22:g.29091178C>A c.1312G>T (p.Asp438Tyr)	rs200050883	missense	VUS	[25,26]

35	***CHEK2***	Chr22:g.29083961C>A c.1556G>T (p.Arg519Leu)	rs587780180	missense	VUS	[27]
36	***MLH1***	Chr3:g.37092003C>G c.2130C>G (p.Asn710Lys)	rs749100096	missense	VUS	[28]
37	***MSH2***	Chr2:g.47672743T>G c.1333T>G (p.Ser445Ala)	rs1553361224	missense	VUS	[29]
38	***MSH2***	Chr2:g.47705617C>T c.2417C>T (p.Thr806Ile)	rs758889557	missense	VUS	[29]
39	***MSH6***	Chr2:g.48025793A>C c.671A>C (p.Glu224Ala)	NR	missense	VUS	NR
40	***MSH6***	Chr2:g.48027683A>T c.2561A>T (p.Lys854Met)	rs34374438	missense	VUS	[30]
41	***MSH6***	Chr2:g.48028063A>G c.2941A>G (p.Ile981Val)	rs730881799	missense	VUS	[28,31]
42	***MSH6***	Chr2:g.48032062C>G c.3452C>G (p.Ala1151Gly)	rs587782625	missense	VUS	NR
43	***MSH6***	Chr2:g.48033693G>A c.3904G>A (p.Ala1302Thr)	rs1553333561	missense	VUS	NR
44	***MSH6***	Chr2:g.48033830-48033831 c.4001+42_4001+45dup	rs587779304	Intronic	VUS	NR
45	***PALB2***	Chr16:g.23647527C>T c.340G>A (p.Gly114Arg)	NR	missense	VUS	NR
46	***PALB2***	Chr16:g.23647304G>C c.563C>G (p.Ala188Gly)	rs587781975	missense	VUS	[32]
47	***PALB2***	Chr16:g.23646872A>T c.995T>A (p.Leu332His)	rs377149139	missense	VUS	NR
48	***PALB2***	Chr16:g.23635372A>C c.2792T>G (p.Leu931Arg)	rs773831304	missense	VUS	[33,34]
49	***PALB2***	Chr16:g.23619215A>G c.3320T>C (p.Leu1107Pro)	rs149194681	missense	VUS	[1,33]
50	***PMS2***	Chr7:g.6043346C>A c.328G>T (p.Ala110Ser)	rs767775907	missense	VUS	[35]
51	***PMS2***	Chr7:g.6042146C>T c.475G>A (p.Val159Met)	rs142416537	missense	VUS	NR
52	***PMS2***	Chr7:g.6038803A>C c.641T>G (p.Val214Gly)	rs864622706	missense	VUS	NR
53	***PMS2***	Chr7:g.6027185G>C c.1211C>G (p.Pro404Arg)	rs536111818	missense	VUS	NR

54	***PMS2***	Chr7:g.6026906C>T<br>c.1490G>A (p.Gly497Asp)	rs199739859	missense	VUS	[36]
55	***PMS2***	Chr7:g.6022617G>A<br>c.2012C>T (p.Thr671Met)	rs587780046	missense	VUS	[37]
56	***RAD51C***	Chr17:g.56774155T>C<br>c.606T>A (p.Asp202Glu)	rs587780256	missense	VUS	NR
57	***RAD51D***	Chr17:g.33428327G>A<br>c.796C>T (p.Arg266Cys)	rs587781813	missense	VUS	[38]
58	***TP53***	Chr17:g.7578262C>T<br>c.587G>A (p.Arg196Gln)	rs483352697	missense	VUS	[39,40]
